# Supplementary material for: AI-driven projection tomography with multicore fibre-optic cell rotation
Source: Nat Commun. 2024 Jan 2;15:147. doi: 10.1038/s41467-023-44280-1 (PMC10762230; doi:10.1038/s41467-023-44280-1)
Supplement: Supplementary file 1 — Supplementary Information LaTeX file [file 41467_2023_44280_MOESM1_ESM.pdf]

# Supplementary Information for AI-driven projection tomography with multicore fibre-optic cell rotation

**Jiawei Sun[1,2,3*], Bin Yang[3], Nektarios Koukourakis[2,3], Jochen Guck[4,5], and Juergen W. Czarske[2,3,6,7*]**

[1]Shanghai Artificial Intelligence Laboratory, Longwen Road 129, Xuhui District, 200232 Shanghai, China
[2]Competence Center for Biomedical Computational Laser Systems (BIOLAS), TU Dresden, Helmholtzstrasse 18, 01069 Dresden, Germany
[3]Laboratory of Measurement and Sensor System Technique (MST), TU Dresden, Germany
[4]Biotechnology Center, Center for Molecular and Cellular Bioengineering, TU Dresden,01307 Dresden, Germany
[5]Max Planck Institute for the Science of Light & Max Planck-Zentrum für Physik und Medizin, 91058 Erlangen, Germany
[6]Cluster of Excellence Physics of Life, TU Dresden, Germany
[7]Institute of Applied Physics, TU Dresden, Germany
[*]Correspondence to: sunjiawei1@pjlab.org.cn, juergen.czarske@tu-dresden.de

**This PDF file includes:**
Supplementary Note 1-5
Supplementary Figure 1-11
Supplementary References 1-16

**Other Supplementary Materials for this manuscript:**
Supplementary Movies 1-3

## Supplementary Note 1. In-situ calibration of the multicore fibre

To achieve dynamic light field modulation through the MCF (FIGH-350S, Fujikura, Japan) for controlled cell rotation, the phase distortion in the MCF, arising from varying phase retardance across fibre cores, needs calibration. In our previous work, we measured this phase delay at the distal end via off-axis digital holography. Applying the conjugated phase distribution to the spatial light modulator (SLM) pre-deforms the wavefront and compensates for the MCF's phase distortion - a technique known as digital optical phase conjugation (DOPC). Following calibration, the reconstructed phase of fibre cores shows that the phase differences for 93.75% of the fibre cores are compensated within the range of $[-\frac{\pi}{8}, \frac{\pi}{8}]$.

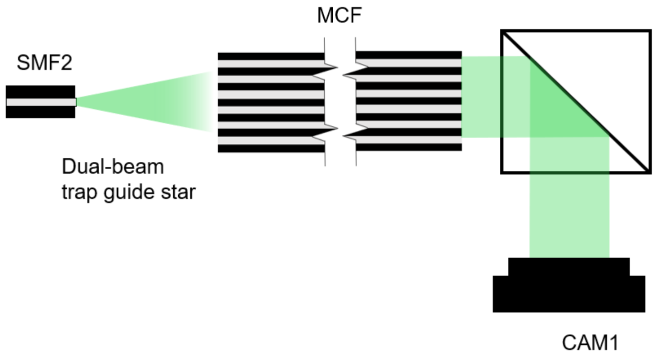


**Supplementary Figure 1.** In-situ calibration of the multicore fibre with one side access. In the multicore fibre-optic cell rotator, the light illuminated from the opposing single-mode fibre is used as the guide star to calibrate the phase distortion in the multicore fibre. CAM1, proximal camera; MCF, multicore fibre; SMF2, single-mode fibre.

However, when integrating the fibres into a microfluidic chip, accessing both MCF ends proves challenging. This necessitates an alternative calibration method that doesn't require distal access. Therefore, we introduce a dedicated in-situ calibration technique for the MCF-OCR. The principle of this approach is illustrated in Supplementary Figure 1. The MCF's light source also couples into the opposing single-mode fibre (SMF) and interferes with the reference beam on the proximal camera (CAM1). The phase distortion in the MCF is subsequently reconstructed from the hologram and compensated by the SLM. This calibration method, compared to the one involving a partial reflector[1], yields sufficient hologram quality and transmission efficiency for precise light field control in the trapping region. However, the spherical wavefront from the emitted beam of the SMF compromises the calibration quality relative to plane wave illumination. Consequently, we employ a multi-modality calibration process for optimal performance. Initially, the phase distortion in MCF is measured using a transmission geometry that requires optical access to both fibre ends. After the integration of the MCF into the MCF-OCR system, any additional phase drifts and temporal or bending phase distortions are in-situ calibrated using this method.

Recent advancements in our lab demonstrate that the phase distortion in MCF can be offset with 3D printed diffractive optical elements on the fibre facet[2,3]. These diffractive optical elements could potentially replace the SLM, paving the way for a less costly, simpler, and more robust setup. Specifically, the previously reported works use the diffractive optical elements for calibrating the phase distortion and generating a focused beam at the MCF output. It remains challenging to create the dynamically modulated light field reported in this work. An alternative approach could be employing a static phase mask to generate a vortex beam for generating the required torque for rotating the cell in the MCF-OCR[4,5]. Also, at the present stage, the calibration quality achieved using diffractive optical elements can hardly reach similar quality compared to using SLMs due to the limited resolution of lithography. Consequently, for optimal calibration, we continue to employ SLMs in this work.

## Supplementary Note 2. Experimental setup

The comprehensive experimental setup of the MCF-OCR tomography system is demonstrated in Supplementary Figure 2. This setup bifurcates into two distinct sections. The optical constituents integral to the MCF-OCR system, delineated by a pink backdrop, are mounted horizontally. Meanwhile, the brightfield microscope, wherein the MCF-OCR is integrated, is marked with a blue background and is mounted vertically.

The optical path starts with the laser beam from a diode-pumped solid-state laser (Laser1; Verdi 532 nm, Coherent Inc.). This beam is split into two paths using a polarizing beamsplitter. The intensity ratio between these two beams can be precisely adjusted by rotating a half-wave plate placed in front of the beamsplitter. One of these beams undergoes an expansion of tenfold in order to fully illuminate the SLM (PLUTO LCOS phase-only SLM, Holoeye Photonics). The computer-generated holograms

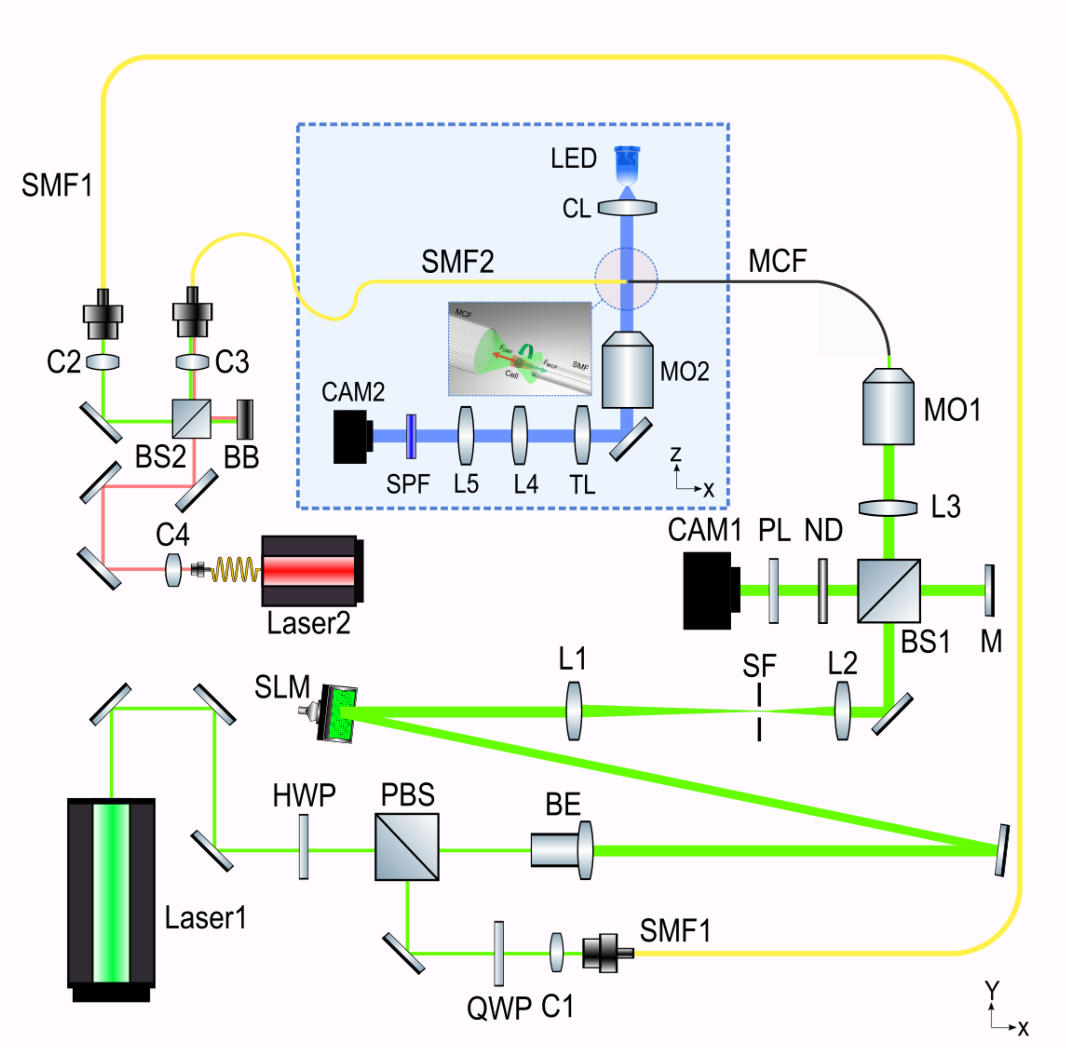


**Supplementary Figure 2.** Schematic representation of the comprehensive experimental setup for the MCF-OCR tomography system, adapted from[6]. BB, beam blocker; BE, beam expander; BS1-2, non-polarizing beamsplitters; C1-4, collimators; CAM1, proximal camera; CAM2, microscope camera; CL, condenser lens; HWP, half-wave plate; L1-5, lenses; LED, light-emitting diode; M, mirror; MO1-2, microscope objectives; ND, neutral density filters; PBS, polarizing beamsplitter; PL, polarizers; QWP, quarter-wave plate; SF, spatial filter; SLM, spatial light modulator; SMF1-2, single-mode fibres; SPF, short pass filter; TL, tube lens.

exhibited on the SLM are composites of a virtual blazed grating, a phase conjugation layer, and a phased array modulation layer. The virtual blazed grating is designed to diffract the phase-modulated beam to the first order. The first diffraction order is subsequently filtered out by the ensuing spatial filter system (L1, SF, L2), thereby eliminating the zero-order term which is the direct reflection from the surface of SLM. The filtered phase modulation hologram is then minified by a factor of 20 and projected onto the proximal fibre facet using a 4-f system comprising an achromatic lens (L3; f=180 mm) and a microscope objective (MO1; 20× Plan Achromat Objective, 0.4 NA, Olympus).

Prior to controlling the light field through the MCF, the phase distortion in the MCF needs to be calibrated via the methods described earlier. The intrinsic phase distortion is measured and compensated in transmission geometry. Once the MCF is integrated into the MCF-OCR system, further phase distortions, specifically those induced by bending and temporal changes, are calibrated employing the opposing SMF ( SMF2; SM600, Thorlabs). Two laser beams are coupled to the SMF2 simultaneously for two distinct purposes. One of the beams, sourced from the near-infrared fibre laser (Laser2; Eylsa 780; Quantel), is employed for optical trapping and manipulation. The second beam is utilized for in-situ calibration of the MCF. This beam is derived from Laser1 and is coupled into another SMF (SMF1; SM400, Thorlabs) using a collimator (C1). In order to eliminate the issue of back-reflected light when coupling the beam into the SMF, a quarter-wave plate (QWP) is employed and rotated to

an angle of 45°. The precise alignment of mirrors and the beamsplitter (BS2) ensures that both beams are efficiently coupled into SMF2 using the collimator (C3). The output beam from the SMF2 maintains a Gaussian beam profile for both wavelengths. During the calibration process, the beam originating from Laser2 is blocked. An off-axis Michelson interferometer (BS1, M) is set up at the proximal side to measure additional phase distortions in the MCF without the need to access the distal side. The off-axis hologram is captured on the proximal camera (CAM1; uEye camera, IDS), and the phase drift in the MCF, induced by bending, is reconstructed from the hologram using the angular spectrum method. This measured phase is conjugated, affine transformed into the coordinate system of the SLM, and then added to the phase conjugation layer of the SLM. This process effectively compensates for the temporal and bending-induced phase distortion, ensuring precise calibration of the phase distortion in the MCF, even after the MCF is bent.

As illustrated in Supplementary Figure 2, a brightfield microscope, designated by a blue background, is utilized to image cell rotation. We employ a blue light-emitting diode (LED; M455L4, Thorlabs, Germany) as an incoherent light source for bright-field imaging. The optical manipulation region is magnified by a microscope objective (50X, 0.42 NA, Mitutoyo), and its projection onto the recording camera (CAM3; Ueye CP, IDS, Germany) is facilitated by an optimized lens system (L4, L5). To remove the scattered light originating from the optical manipulation beams, we mount short-pass filters (FES0500, Thorlabs, Germany) in front of the camera. These filters greatly enhance the clarity and contrast of the projected images, thus ensuring accurate tomographic reconstruction.

## Supplementary Note 3. Dynamic light field control using deep learning

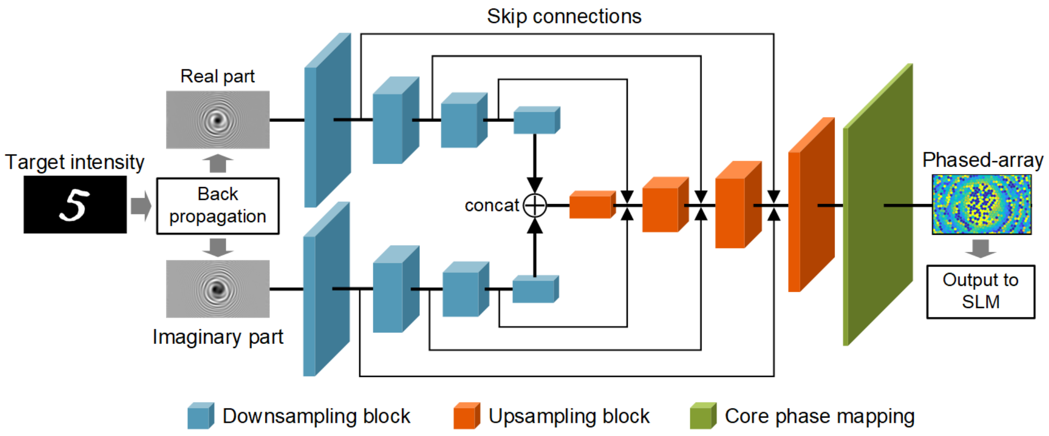


**Supplementary Figure 3.** The architecture of the physics-informed phase encoder deep neural network (CoreNet) utilized for hologram generation. CoreNet is trained using a self-supervised learning approach based on the diffraction propagation model.

Accurate and high-fidelity rapid light field control within the MCF-OCR is imperative to precisely manipulate optical forces required for cell rotation. This control is ensured through the computation of holograms using a previously proposed physics-informed deep neural network - CoreNet at near video rates, thereby facilitating high-fidelity beam control within the MCF-OCR[7]. The CoreNet's architecture is illustrated in Supplementary Figure 3. Given that the input image (target intensity distribution in the far field) and the desired output image (phase modulation hologram) are distinct modalities, we initially employ the diffraction model to back-propagate the target intensity image to the same physical imaging plane. This is due to the simplified feature mapping at the same plane. The real part, indicating the light field amplitude, and the imaginary part, symbolizing the phase of the computed complex value, are incorporated as dual physical prior inputs into the network. The downsampling blocks are replicated for each input, with the two downsampling paths converging at a concatenation layer via the addition operator. The output from this layer is then up-sampled to the size of a CGH by a series of upsampling blocks.

Each downsampling and upsampling block comprises two residual blocks. Each of these residual blocks includes two sets of batch normalization, a nonlinearity (ReLU), and a convolutional layer. The strides of the first convolution and transposed convolution in the residual block are set to (2,2) for downsampling and upsampling. Furthermore, the experimentally determined MCF core distribution map is applied to the network output, producing the tailored phase modulation map for the phased array. Utilizing a self-supervised learning method precludes the need for a preliminary calculation of the ground truth phase modulation map for each input target intensity image. Instead, the phase modulation map is numerically propagated to the target plane using the diffraction model, thereby reconstructing the target intensity image within the model. The loss function is

represented by the negative Pearson correlation coefficient (NPCC), as defined in Eq. (1), between the CoreNet reconstructed target intensity and the input target intensity.

$$\mathscr{L}_{\mathrm{NPCC}}(X,Y) = (-1) \times \frac{\sum_{i}^{n} (X_i - \bar{X})(Y_i - \bar{Y})}{\left\{ \sum_{i}^{n} (X_i - \bar{X})^2 \sum_{i}^{n} (Y_i - \bar{Y})^2 \right\}^{1/2}} \tag{1}$$

The NPCC quantifies the linear correlation between two images as opposed to computing pixel-wise errors, thus enhancing the likelihood of convergence for intricate images. During the training process, this loss value is retroactively fed into the encoder section via the Adam optimizer, leading to the update of the CoreNet's learnable parameters. The Network was trained on a workstation with Xeon CPU E5-2650 (2.20 GHz) and 128 GB of RAM, using NVIDIA Quadro GV100 GPU. Owing to the physics-informed model in the network architecture, convergence is achieved after a relatively brief training period of just five epochs on the MNIST dataset. These images were resized to a dimension of $512 \times 512$ pixels, and subsequently padded with zeros to achieve a size of $1920 \times 1080$ pixels. Consequently, the CoreNet produced corresponding modulation holograms, which were then promptly transmitted to the Spatial Light Modulator (SLM). Remarkably, CoreNet slashed the computation time to 0.14 seconds per hologram. This substantial reduction has effectively enabled real-time, adaptive light-field control at the far field of the MCF, with a frame rate of 7.1 frames per second.

## Supplementary Note 4. Autonomous tomographic reconstruction workflow

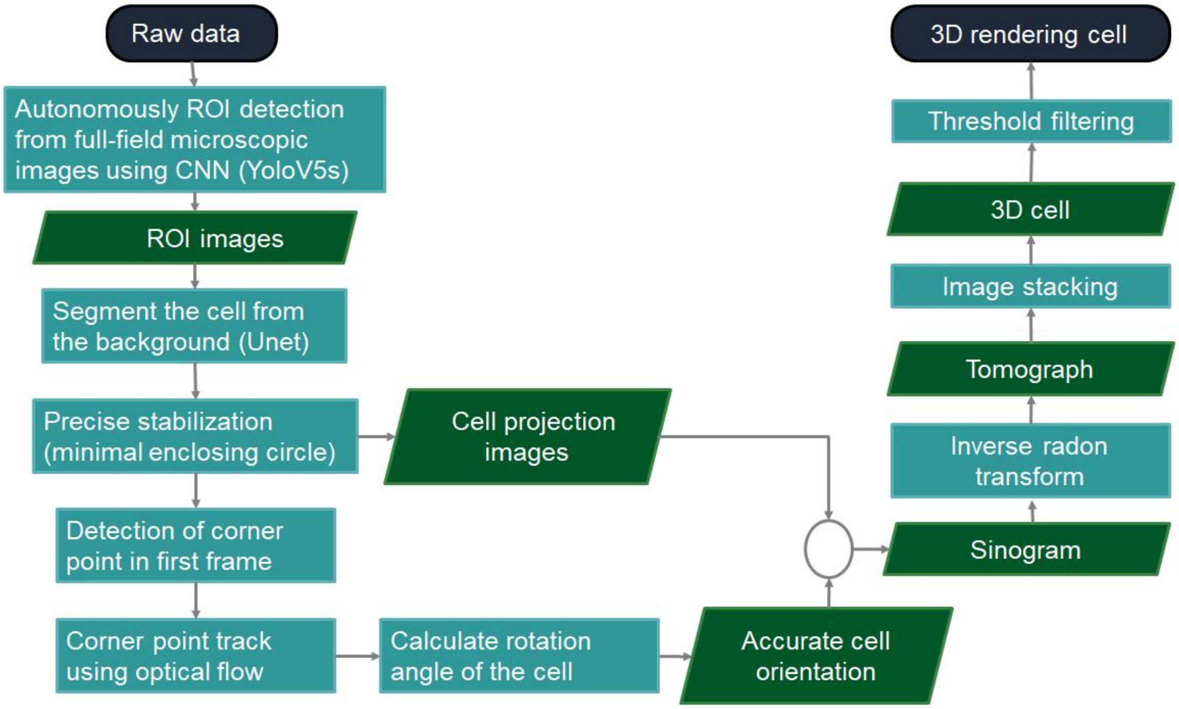


**Supplementary Figure 4.** Comprehensive flow chart of the autonomous tomographic reconstruction workflow for reconstructing the raw cell rotation video to the 3D intensity distribution.

The comprehensive flow chart of the AI-driven autonomous tomographic reconstruction is presented in Supplementary Figure 4.

### Cell detection using YOLOv5

In the initial phase of our proposed workflow, we employ an object detection neural network to locate the position of the cell in the given image to determine the region of interest rapidly for reducing the raw data size. Object detection neural networks

find broad applications across a myriad of fields, notably in autonomous driving and face recognition. YOLOv5, an acronym for "You Only Look Once version 5"[8], is a pre-trained model recognized for its proficiency in performing object detection tasks within the field of computer vision. This iteration of the YOLO series accentuates its predecessors by introducing salient enhancements in performance, usability, and computational efficiency. YOLOv5 is heralded for its speed and efficiency, thus rendering it appropriate for applications necessitating immediate object detection. Concurrently, it does not compromise on accuracy, employing sophisticated mechanisms such as multiple bounding box predictions per grid cell and class probability. The smallest and fastest variant of the YOLOv5 - YOLOv5s is chosen due to its high efficiency and compact model size. The pre-trained YOLOv5s are already capable of identifying a variety of general features in images due to initial training on a large-scale dataset. When applying it to our task of localizing the cells, we use transfer learning to update the weights in the model for better performance in our task.

The detection of cells in our study hinges on the transfer learning capabilities of YOLOv5, a state-of-the-art convolutional neural network (CNN) model known for its proficiency in object detection tasks. Our target, the cell, constitutes a specific object type that can be accurately isolated and recognized through the YOLOv5 model.

Transfer learning refers to the process where a pre-trained model is repurposed and fine-tuned on a different but related problem. The YOLOv5 model was initially trained on a large-scale, diverse dataset, equipping it with the capability to identify a variety of general features in images. When introduced to the task of cell detection, it does not need to initiate learning from scratch. Instead, it adapts its pre-existing knowledge, updating weights according to the new data. This ability to transfer knowledge from one domain to another substantially expedites the training process and still achieves high performance, particularly in scenarios where labeled data is limited. In our task, 100 manually labeled cell images served as the basis for model training. To enhance the robustness of the model and simulate different conditions, these labeled images underwent extensive augmentation processes, including rotation and random cropping. These operations expanded our training dataset to 2000 images for avoiding overfitting.

The Generalized Intersection over Union (GIoU) loss is a modification of the standard Intersection over Union (IoU) metric used in transfer learning of the YOLOV5. GIoU not only considers the overlapping area between the predicted bounding box and the ground truth bounding box (like IoU), but it also considers the area of the smallest enclosing box that contains both the predicted and the ground truth boxes. The GIoU loss $L_{GIou}$ is defined as

$$L_{\mathrm{GIoU}} = 1 - \frac{\mathrm{Area}(B_{\mathrm{pred}} \cap B_{\mathrm{true}})}{\mathrm{Area}(B_{\mathrm{pred}} \cup B_{\mathrm{true}})} + \frac{\mathrm{Area}(C) - \mathrm{Area}(B_{\mathrm{pred}} \cup B_{\mathrm{true}})}{\mathrm{Area}(C)} \tag{2}$$

where $B_{\mathrm{pred}}$ is the predicted bounding box, $B_{\mathrm{true}}$ is the ground truth bounding box, $C$ is the smallest enclosing box that contains both $B_{\mathrm{pred}}$ and $B_{\mathrm{true}}$ $\mathrm{Area}(A)$ is a function that calculates the area of box $A$. The $\cap$ operator denotes intersection of two boxes. The $\cup$ operator denotes the total area covered by both boxes.

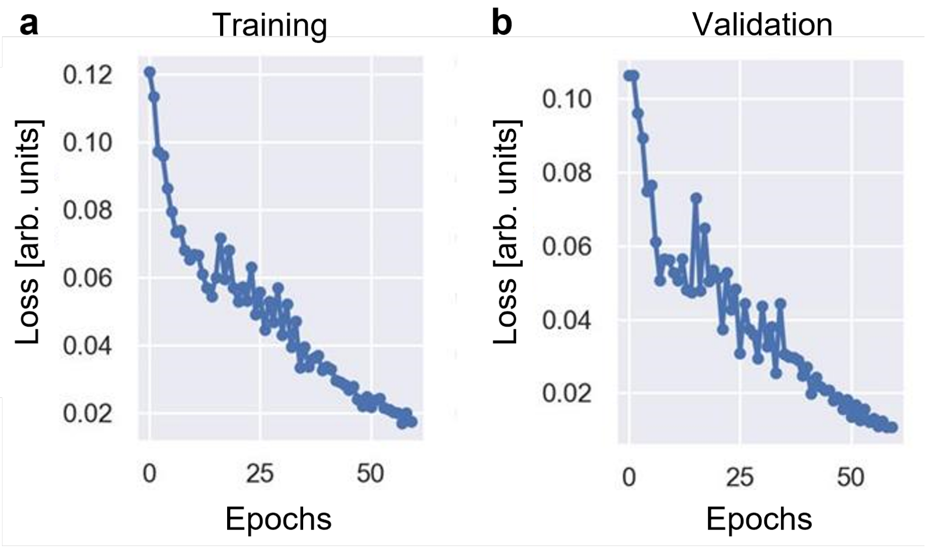


**Supplementary Figure 5.** (a) Training loss curve and (b) Validation loss curve for the YOLOv5 model trained over the course of 60 epochs. Arb. units, arbitrary units.

The calculated loss for the training and validation are shown in Supplementary Figure 5. The training phase of the model was observed to reach convergence after training of 60 epochs. At this point, the changes in the loss function became negligible, indicating that the model had learned the features necessary for cell detection from the training data. The convergence of the model demonstrates that the transfer learning and data augmentation strategies employed were effective for this cell detection task. This enables robust real-time detection of the cell from the microscopic images.

## Learning-based cell segmentation

Image segmentation has a great influence on the performance of tomographic reconstruction. Effective segmentation techniques can help to distinguish the biological sample, from the noise present in the background. As shown in the Figure 6 from the main text, the contrast of the final 3D reconstruction is significantly improved using image segmentation techniques.

Typically, edge detection algorithms such as the Canny filter[9], Sobel filter[10], and Hough transform[11] have been employed to determine the boundaries of cells in microscopic images. These methods have been effective for a multitude of applications, given their ability to capture gradient changes along object borders. However, their performance is dependent upon precise parameter tuning, which varies for different samples and conditions. Moreover, these traditional methods rely heavily on high contrast between the object and the background, a condition that cannot always be fulfilled, particularly in the imaging of biological samples. For instance, in our study, we encounter scenarios where the contrast between the cell and its background is not sufficient for effectively employing these classical algorithms. For 3D tomographic reconstruction, uniform segmentation across all frames is required, which can be a tedious task given the need for fine-tuning parameters for traditional algorithms. This is where deep learning-based methods, with their inherent feature-learning ability, have been found advantageous. They offer consistent, homogeneous segmentation across different frames without the need for meticulous parameter adjustments[12].

Among the various deep learning architectures, the U-Net architecture has emerged as a powerful tool for image segmentation tasks[13]. Its design is simple yet effective, making it ideal for biomedical image segmentation. Given its ability to learn complex patterns from a limited amount of data, we employ a pre-trained U-Net network for our cell segmentation task. A total of 215 manually labeled images were used to fine-tune this pre-trained network, allowing it to adapt its learning to our specific task of single cell-background segmentation. The network exhibited exceptional performance, clearly segregating the cells from the background, thus paving the way for high-quality tomographic reconstruction.

## Precise stabilization using minimal enclosing circle

After the cell segmentation, there are still minor offsets of the cell in different frames, which means the centre of the cell in different frames is at different points. To calibrate these offsets, we employ the minimal enclosing circle method. This identifies the smallest circle that completely encompasses the cell in each frame. Thus, the precise position of the cell is determined by the centre coordinate of the minimal enclosing circle. By adjusting the frames accordingly, we ensure the cell maintains a consistent position in the video, achieving refined stabilization as shown in Supplementary Figure 6.

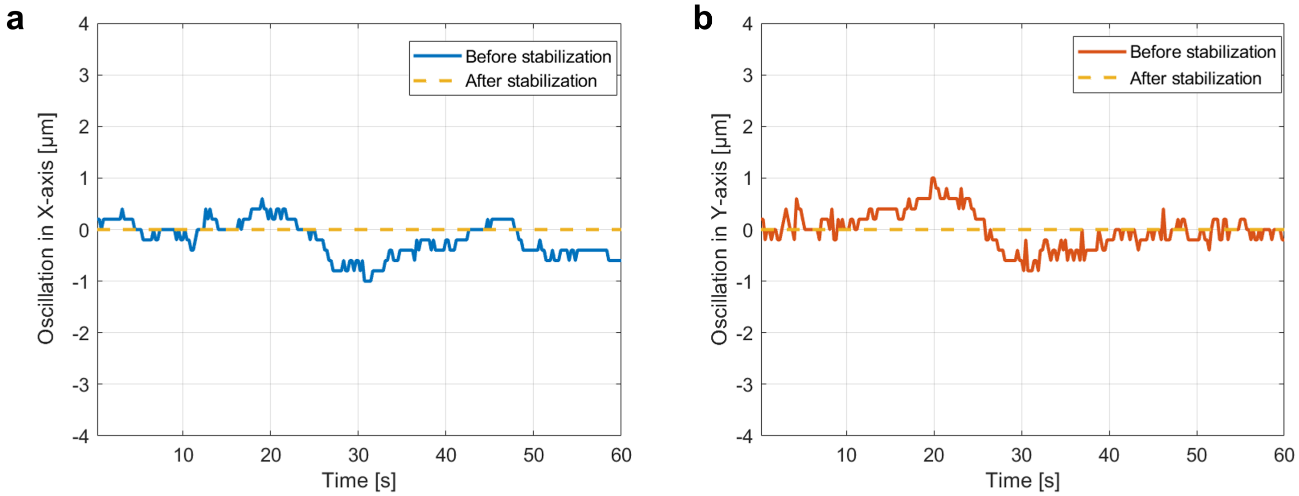


**Supplementary Figure 6.** Quantitative comparison of cell position in (a) X-axis (b) Y-axis before and after implementing the stabilization process.

## Automatic detection of the cell rotation angle

Accurate detection of the rotation angles is crucial for maintaining the fidelity of tomographic reconstructions. Thus the ability to track cell orientation automatically within microscopic images presents a significant advantage. However, accomplishing this task remains challenging due to the imaged cellular structures typically having complex and heterogeneous internal structures. These intricacies can become indistinct or completely obscured due to the inherent resolution limitations of optical microscopy and the natural transparency of cells. As a result, discerning the rotation angle solely based on these often subtle and potentially obscured structures is no trivial endeavor. Furthermore, the process of cell rotation adds an additional layer of complexity to the task of orientation tracking. Throughout the rotation, different portions of the cell cyclically come into view and subsequently disappear from the field of view. This dynamic alteration of visible cell parts can confound attempts at consistent tracking, making the detection of the rotation angle a complex and challenging problem. Despite these obstacles, the development of effective solutions to accurately track cell orientation throughout rotation remains an active area of research, with potential applications in enhancing the quality of tomographic reconstructions.

Here, we implement Harris corner detector[14,15] to achieve dynamic feature extraction on the cell rotation video. The algorithm works by detecting large variations in the gradient direction of edges around each pixel in the image. This is used to identify points in a digital image where the direction of edges changes significantly. The detector approximates the local autocorrelation function of the image, thereby determining the "cornerness" of a region.

Consider a window in a grayscale image centered at position (x, y), with pixel grayscale value $I(x,y)$. If the window moves by a small displacement $u$ and $v$ in the x and y direction respectively, to a new position $(x+u, y+v)$, the pixel grayscale value at the new position will be $I(x+u, y+v)$. The difference $[I(x+u, y+v) - I(x,y)]$ represents the change in grayscale value due to the movement of the window.

We introduce a window function $\omega(x,y)$ at the position $(x,y)$, representing the weight of each pixel in the window. The simplest choice is to set the weight of all pixels in the window to 1, though sometimes it's chosen as a Gaussian function centered at the window center. The change in pixel grayscale value due to the window moving in all directions can then be expressed as:

$$E(u,v) = \sum_{(x,y)} \omega(x,y) \times [I(x+u, y+v) - I(x,y)]^2 \tag{3}$$

For a corner, this function results in a large value. Therefore, to identify corners in the image, we can seek to maximize this function. Direct calculation of $E(u,v)$ using Supplementary Equation 3 is computationally demanding, so we instead use a first-order Taylor expansion to approximate this function:

$$I(x+u, y+v) \approx I(x,y) + uI_x + vI_y \tag{4}$$

where $I_x = \frac{\partial I(x,y)}{\partial x}$ and $I_y = \frac{\partial I(x,y)}{\partial y}$ are the gradients in the x and y directions, respectively. Substituting the above approximation into Supplementary Equation 3, we get:

$$\begin{aligned} E(u,v) &= \sum_{(x,y)} \omega(x,y) \times [I(x,y) + uI_x + vI_y - I(x,y)]^2 \\ &= \sum_{(x,y)} \omega(x,y) \times [uI_x + vI_y]^2 \\ &= \sum_{(x,y)} \omega(x,y) \times [u^2 {I_x}^2 + v^2 {I_y}^2 + 2uvI_xI_y] \end{aligned} \tag{5}$$

This can be expressed in matrix form:

$$E(u,v) \approx \begin{bmatrix} u & v \end{bmatrix} M \begin{bmatrix} u \\ v \end{bmatrix} \tag{6}$$

where the matrix $M$ is defined as:

$$M = \sum_{(x,y)} \omega(x,y) \begin{bmatrix} {I_x}^2 & I_xI_y \\ I_xI_y & {I_y}^2 \end{bmatrix} \rightarrow R^{-1} \begin{bmatrix} \lambda_1 & 0 \\ 0 & \lambda_2 \end{bmatrix} R \tag{7}$$

The orthogonal transformation, represented by matrix $R$, is performed for diagonalization of the real symmetric matrix $M$. This matrix $R$ serves as a rotation factor that leaves invariant the variation components along two orthogonal directions. The process of diagonalization helps in revealing these components, namely $\lambda_1$ and $\lambda_2$, which are the eigenvalues of the matrix $M$. They represent the magnitude of changes in the two orthogonal directions in the image. These eigenvalues provide a quantifiable measure of corner-like features, forming the basement of the Harris corner detection algorithm. The magnitude and relation of these eigenvalues provide insightful information about the local image structure, allowing the distinction between flat regions, edges, and corners.

The aim of the algorithm is to identify the windows that produce significant variations in pixel intensity when displaced. The magnitude of these variations depends largely on the structure of the matrix $M$. To find these windows, we can utilize the eigenvalues of $M$. The function $E(u,v)$ is defined as the difference between the determinant of $M$ and the square of the trace of $M$, scaled by an empirical constant $k$:

$$E(u,v) = \det(M) - k(\mathrm{trace}(M))^2 \tag{8}$$

where the determinant of matrix $M$ is the product of its eigenvalues $\lambda_1$ and $\lambda_2$:

$$\det(M) = \lambda_1 \lambda_2 \tag{9}$$

And the trace of matrix $M$ is the sum of its eigenvalues $\lambda_1$ and $\lambda_2$:

$$\mathrm{trace}(M) = \lambda_1 + \lambda_2 \tag{10}$$

Here, the constant $k$ is an empirical value, typically set around 0.05. The eigenvalues $\lambda_1$ and $\lambda_2$ are the principal indicators of local image structure, allowing the detection of flat regions, edges, and corners.

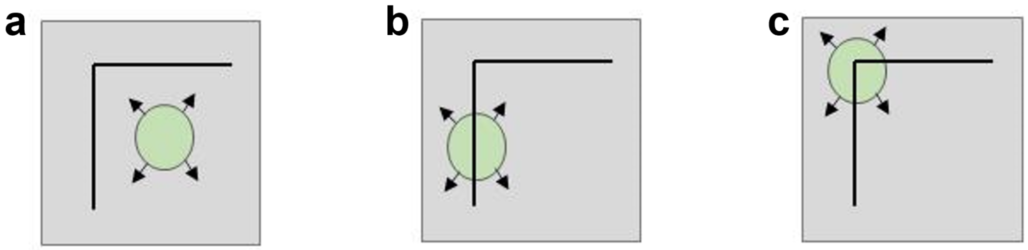


**Supplementary Figure 7.** Principle of the Harris Corner Detector. The illustration shows the detection of corner points, which are intersections of two edges and represent locations of significant changes in edge directions. A moving window (depicted as the green circle) scans the region of interest. (a) In flat regions, no changes in the gradient map amplitude are observed irrespective of the window's direction. (b) In edge regions, the amplitude of the gradient map remains constant along the edge directions but varies across them. (C) In corner regions, the gradient map's amplitude exhibits significant changes in all directions, thereby identifying the presence of a corner.

The response of the window function to the local structure of the image can be characterized according to the magnitudes of the eigenvalues, $\lambda_1$ and $\lambda_2$. As shown in Supplementary Figure 7a, in flat regions of the image with uniform intensity, the window function traverses an area of uniform grayscale values. Consequently, the changes in the grayscale value along both horizontal and vertical directions are minimal, implying small values for both partial derivatives $I_x$ and $I_y$. This results in the eigenvalues $\lambda_1$ and $\lambda_2$ being small, yielding a low response value $R$. As demonstrated in Supplementary Figure 7b, along an edge in the image, significant intensity variation is observed in one direction (either horizontal or vertical), while the variation is minimal in the orthogonal direction. This is reflected by one partial derivative being considerably larger than the other, i.e., $\lambda_1 >> \lambda_2$ or $\lambda_2 >> \lambda_1$. The response $R$ becomes negative in such cases, indicating the presence of an edge. As illustrated in Supplementary Figure 7c, at corner points, the image intensity varies significantly in both horizontal and vertical directions. Hence, both partial derivatives, $I_x$ and $I_y$, are large, leading to large eigenvalues $\lambda_1$ and $\lambda_2$. This results in a high response value $R$, indicating the presence of a corner. Hence, by examining the eigenvalues of the Harris Matrix, we can discern whether a given window within the image contains a plane, an edge, or a corner, allowing us to extract key features from the image.

Once the features are identified using the Harris corner detector, the next step is to track their movement across the image sequence, allowing for the calculation of the cell's rotation angle. The Lucas-Kanade optical flow method is a widely employed approach in feature movement estimation in video[16]. Optical flow pertains to the apparent motion of brightness patterns in an image, and it can be represented as a 2D velocity field associated with the spatial movement of intensity patterns over time. The optical flow problem is essentially framed as an image brightness constancy assumption, implying that the intensity of a given image pixel remains consistent over time. This assumption yields the fundamental optical flow equation:

$$I(x,y,t) = I(x+u, y+v, t+1) \tag{11}$$

where $I(x,y,t)$ denotes the intensity distribution of the image at a given pixel position $(x,y)$ and time $t$. $u$ and $v$ signify the horizontal and vertical components of the optical flow. Rearranging and approximating this equation using a Taylor series expansion leads to the basic optical flow equation

$$I_x u + I_y v + I_t = 0 \tag{12}$$

where $I_x$ and $I_y$ are the spatial intensity gradients, and $I_t$ is the temporal intensity gradient. The Lucas-Kanade method solves this equation for $u$ and $v$ under the assumption that the displacement of the intensity pattern is small and approximately constant within a neighborhood window around each pixel. This transforms the problem into an overdetermined system of linear equations that can be expressed in matrix form

$$\begin{bmatrix} \sum I_x I_x & \sum I_x I_y \\ \sum I_x I_y & \sum I_y I_y \end{bmatrix} \begin{bmatrix} u \\ v \end{bmatrix} = \begin{bmatrix} -\sum I_x I_t \\ -\sum I_y I_t \end{bmatrix} \tag{13}$$

This can be solved for the optical flow vectors $[u,v]$ via least squares. By solving the equation for the local neighborhood around each tracked feature on the rotated cell, the Lucas-Kanade method estimates the motion vector, which represents the displacement of the tracked feature between consecutive frames. The set $W$ represents the neighborhood of pixels around the feature $(x_0, y_0)$, which is defined as

$$W = \left\{ (i,j) | \sqrt{(i-x_0)^2 + (j-y_0)^2} < k \right\} \tag{14}$$

where $k$ is the radius of the window. Therefore, the shift of the feature in X-axis $\Delta x$ and Y-axis $\Delta y$ can be defined as

$$\Delta x = u = \frac{\sum_{(i,j)\in W} I_x(i,j) I_t(i,j)}{\sum_{(i,j)\in W} I_x(i,j)^2 + I_y(i,j)^2}, \tag{15}$$

$$\Delta y = v = \frac{\sum_{(i,j)\in W} I_y(i,j) I_t(i,j)}{\sum_{(i,j)\in W} I_x(i,j)^2 + I_y(i,j)^2}. \tag{16}$$

Assuming the cell is a perfect sphere with a radius of $R$, the rotation angle of the cell around the X-axis can be calculated by

$$\theta_x = \arcsin \frac{\Delta y}{R} \tag{17}$$

and Y-axis rotation angle

$$\theta_y = \arcsin \frac{\Delta x}{R} \tag{18}$$

### Tomographic reconstruction

The procedure for tomographic reconstruction is classified into three sequential stages: sinogram generation, tomograph creation, and three-dimensional stacking.

In the first stage, a sinogram is generated, illustrated in Supplementary Figure 8, which is a unique form of image that represents the radon transform of an object's cross-sectional layers. Each line of the sinogram corresponds to a different

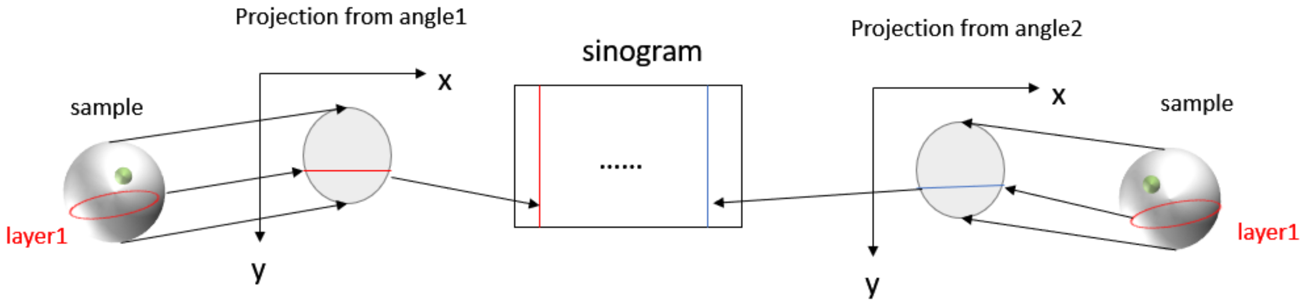


**Supplementary Figure 8.** Illustration of the sinogram principle. The red circle represents the 2D cross-sectional layer of the 3D sample. Two projections on the left and right correspond to sample data at 'angle1' and 'angle2' respectively. In the left projection, a distinct red horizontal line signifies the specific projection of the chosen layer, 'layer1', at 'angle1'. On the right, a blue horizontal line represents 'layer1' at 'angle2'. The sinogram is a composition created by collating these individual layer projections taken from different angles into a single image, effectively providing a comprehensive view of the layer from multiple perspectives.

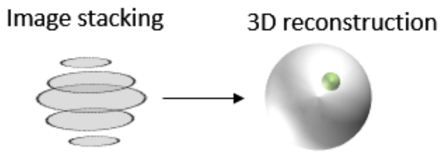


**Supplementary Figure 9.** The 3D reconstruction of the sample can be obtained by stacking the 2D cross-sectional layers.

projection angle, and every pixel value in that line represents the total attenuation along a ray at that angle. The generation of the sinogram involves compiling projections from various angles of a given layer of the sample. These projections are then assembled into a unique image configuration, known as a sinogram.

Once the sinogram is generated, it serves as input data for the inverse Radon transform, a mathematical operation used in computed tomography to convert the sinogram back into the image. The transformation yields a tomograph, which represents a reconstructed image of each layer of the object. The accuracy of the reconstruction is influenced by factors such as the number of projections, the angular range, and the noise level. In the final stage of the tomographic reconstruction process, as illustrated in Supplementary Figure 9, all the reconstructed cross-sectional slices, known as tomography, are stacked to form a 3D model. This comprehensive model allows for the detailed visualization and analysis of the sample's internal structural integrity and features from various perspectives.

## Supplementary Note 5. Validation of the AI-driven tomographic reconstruction workflow using an artificial cell phantom

To validate the precision and robustness of our AI-driven tomographic reconstruction workflow, particularly when applied to real experimental data, we employed an artificial cell phantom. The artificial cell phantom was trapped in a dual-beam fibre-optic trap and rotated by the microfluid control. This serves not only as a validation to the accuracy of our approach but also establishes its versatility across different cell rotation tomography systems. The artificial cell phantom was manufactured using a transparent hydrogel bead and two silica microbeads embedded with a diameter of 2.9 µm and 2.5 µm respectively[17].

The 3D intensity distribution of this artificial cell phantom, as reconstructed through our AI-enhanced workflow, is demonstrated in Supplementary Figure 10. In the reconstructed 3D intensity distribution, both microbeads are distinctly discernible across the XY, YZ, and XZ planes, validating the high fidelity of our tomographic reconstruction process. Furthermore, careful observation of the hydrogel's boundary reveals subtle textural variations, indicative of regions where the boundary material has been displaced due to the presence of the embedded microbeads. These intricacies are especially obvious in Supplementary Figure 10c. Overall, the microbeads compared to the hydrogel is clearly visible and the structure of the phantom is well-resolved

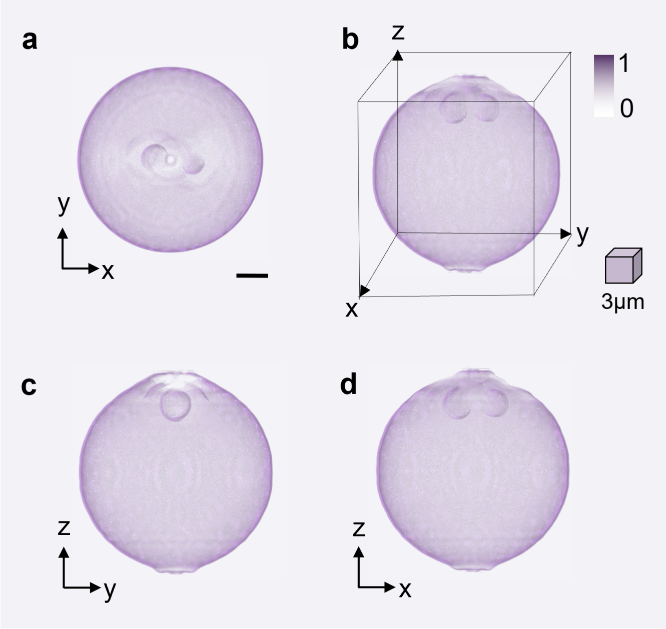


**Supplementary Figure 10.** 3D intensity distribution reconstruction of the artificial cell phantom containing two microbeads. (a)Top-down view along the Z-axis, scale bar 3 µm. (b) Isometric view at an angle of 45°, scale cube 3×3×3 µm$^3$. (c) Lateral view aligned with the X-axis. (d) Lateral view aligned with the Y-axis.

in the 3D reconstruction.

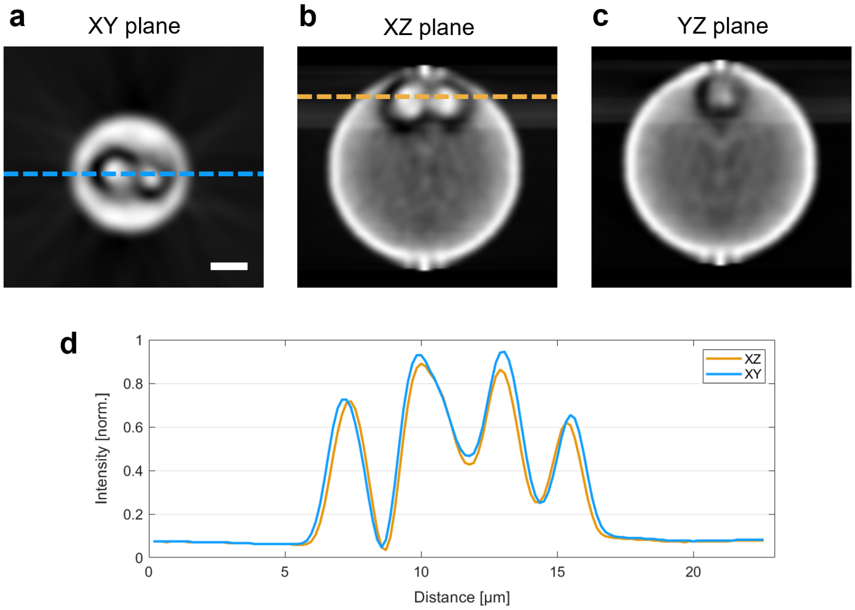


**Supplementary Figure 11.** Cross sections of the reconstructed artificial cell phantom at (a) XY plane (b) XZ plane (c) YZ plane, scale bar 3 µm. (d) Intensity profile along the colour marked lines in XY plane (blue) and XZ plane (yellow).

To conduct a quantitative assessment of the tomographic reconstruction across various 3D orientations, we present cross-sectional images of the tomograph in the XY, XZ, and YZ planes, as depicted in Supplementary Figure 11a-c. The intensity profiles of the two microbeads, illustrated in Supplementary Figure 11d, demonstrating a high contrast between the silica microbead and its hydrogel surroundings. Moreover, the resolution achieved in XY and XZ planes is closely aligned, indicating a near-isotropic resolution in the 3D space.

## Supplementary References


1. Kuschmierz, R., Scharf, E., Koukourakis, N. & Czarske, J. W. Self-calibration of lensless holographic endoscope using programmable guide stars. *Opt. letters* **43**, 2997–3000 (2018).
2. Kuschmierz, R., Scharf, E., Ortegón-González, D. F., Glosemeyer, T. & Czarske, J. W. Ultra-thin 3d lensless fiber endoscopy using diffractive optical elements and deep neural networks. *Light. Adv. Manuf.* **2**, 1–10 (2021).
3. Dremel, J., Scharf, E., Kuschmierz, R. & Czarske, J. Minimal-invasive faseroptische endomikroskopie für die medizin. *tm-Technisches Messen* **89**, 25–30 (2022).
4. Arrizón, V., Ruiz, U., Sánchez-de-la Llave, D., Mellado-Villaseñor, G. & Ostrovsky, A. S. Optimum generation of annular vortices using phase diffractive optical elements. *Opt. letters* **40**, 1173–1176 (2015).
5. Simpson, N., Dholakia, K., Allen, L. & Padgett, M. Mechanical equivalence of spin and orbital angular momentum of light: an optical spanner. *Opt. letters* **22**, 52–54 (1997).
6. Sun, J., Koukourakis, N. & Czarske, J. W. Complex wavefront shaping through a multi-core fiber. *Appl. Sci.* **11**, 3949 (2021).
7. Sun, J. *et al.* Real-time complex light field generation through a multi-core fiber with deep learning. *Sci. reports* **12**, 1–10 (2022).
8. Redmon, J., Divvala, S., Girshick, R. & Farhadi, A. You only look once: Unified, real-time object detection. In *Proceedings of the IEEE conference on computer vision and pattern recognition*, 779–788 (2016).
9. Xu, Z., Baojie, X. & Guoxin, W. Canny edge detection based on open cv. In *2017 13th IEEE international conference on electronic measurement & instruments (ICEMI)*, 53–56 (IEEE, 2017).
10. Aqrawi, A. A. & Boe, T. H. Improved fault segmentation using a dip guided and modified 3d sobel filter. In *SEG Technical Program Expanded Abstracts 2011*, 999–1003 (Society of Exploration Geophysicists, 2011).
11. Illingworth, J. & Kittler, J. A survey of the hough transform. *Comput. vision, graphics, image processing* **44**, 87–116 (1988).
12. Wang, S., Yang, D. M., Rong, R., Zhan, X. & Xiao, G. Pathology image analysis using segmentation deep learning algorithms. *The Am. J. Pathol.* **189**, 1686–1698, DOI: https://doi.org/10.1016/j.ajpath.2019.05.007 (2019).
13. Ronneberger, O., Fischer, P. & Brox, T. U-net: Convolutional networks for biomedical image segmentation. In *International Conference on Medical image computing and computer-assisted intervention*, 234–241 (Springer, 2015).
14. Harris, C., Stephens, M. *et al.* A combined corner and edge detector. In *Alvey vision conference*, vol. 15, 10–5244 (Citeseer, 1988).
15. Gao, X., Xiao, B., Tao, D. & Li, X. Image categorization: Graph edit distance+ edge direction histogram. *Pattern Recognit.* **41**, 3179–3191 (2008).
16. Decarlo, D. & Metaxas, D. Optical flow constraints on deformable models with applications to face tracking. *Int. J. Comput. Vis.* **38**, 99–127 (2000).
17. Schürmann, M. *et al.* Three-dimensional correlative single-cell imaging utilizing fluorescence and refractive index tomography. *J. biophotonics* **11**, e201700145 (2018).
