# Supplementary material for: AI-driven projection tomography with multicore fibre-optic cell rotation
Source: Nat Commun. 2024 Jan 2;15:147. doi: 10.1038/s41467-023-44280-1 (PMC10762230; doi:10.1038/s41467-023-44280-1)
Supplement: Supplementary file 3 — Description of Additional Supplementary Files [file 41467_2023_44280_MOESM3_ESM.pdf]

Description of Additional Supplementary Files

Supplementary Movie 1
Description: Working principle of the multicore fibre-optic cell rotator (MCF-OCR) tomography system.

Supplementary Movie 2
Description: Validation of the AI-driven workflow utilizing a cell phantom.

Supplementary Movie 3
Description: Visual comparison of three-dimensional intensity distribution reconstruction for a live HL60 human leukaemia cancer cell using different methods.
